# The association between glycated hemoglobin levels and in-stent restenosis following percutaneous coronary intervention in coronary artery disease patients

**DOI:** 10.3389/fendo.2026.1793093

**Published:** 2026-04-23

**Authors:** Yuyang Lei, Ping Jin, Hui Liu, Lin Su, Di Wu, Chenxi Sun, Haoyu Wu, Juan Zhou, Chen Wang

**Affiliations:** 1 Department of Cardiovascular Medicine, First Affiliated Hospital of Xi’an Jiaotong University, Xi’an, Shaanxi, China; 2 Department of Cardiology, Second Affiliated Hospital of Xi’an Jiaotong University, Xi’an, Shaanxi, China; 3 Biobank, First Affiliated Hospital of Xi’an Jiaotong University, Xi’an, Shaanxi, China

**Keywords:** coronary artery disease, generalized additive model (GAM), glycated hemoglobin, in-stent restenosis, two-piecewise linear model

## Abstract

**Background:**

The association between glycated hemoglobin (HbA1c) and in-stent restenosis (ISR) in coronary artery disease (CAD) patients following percutaneous coronary intervention (PCI) is not well understood. We examined the association between HbA1c levels and ISR risk among CAD patients after PCI.

**Methods:**

We conducted a retrospective study of 6297 CAD patients following PCI. Patients were stratified into three groups based on admission HbA1c levels. The primary outcome was the incidence of ISR. Adjusted logistic regression models were used to assess the association between HbA1c levels and ISR. We employed the generalized additive model (GAM) to examine potential relationships, with subsequent stratified analyses to evaluate the robustness of the main results and to test for effect modification by additional clinical variables.

**Results:**

Among this study participants, a total of 1305 individuals were diagnosed with ISR, representing 20.72% of the cohort. The distribution of participants across HbA1c categories, along with corresponding sample sizes and proportions, was as follows: HbA1c <5.7%, 914 individuals (14.51%). HbA1c ≥5.7% to <6.5%, 2783 individuals (44.20%). HbA1c ≥6.5%, 2600 individuals (41.29%). After controlling for confounders, higher HbA1c levels were significantly linked to a greater risk of ISR [OR:1.18, 95%CI (1.09–1.27), p <0.0001]. Specifically, individuals with HbA1c ≥6.5% exhibited a substantially higher risk [OR:1.70, 95% CI (1.26–2.29), p =0.0005]. The GAM analysis revealed a non-linear association between HbA1c levels and the risk of ISR. The threshold effect analysis revealed a turning point at an HbA1c level of 7.8%. Below this threshold, the association between HbA1c and ISR risk was more pronounced.

**Conclusion:**

This study showed a non-linear relationship between increased HbA1c levels and the risk of ISR in CAD patients post-PCI.

## 1 Introduction

Coronary artery disease (CAD) lies at the global burden of disease and premature death, accounting for roughly 30% of the global cardiovascular disease burden (1, 2). Stent-based percutaneous coronary intervention (PCI) is fundamental in the initial treatment of acute coronary syndrome (ACS) patients (3). Although stent technology has advanced considerably, in-stent restenosis (ISR) remains major clinical challenge, leading to severe cardiovascular complications and negative patient outcomes (4, 5). Early detection of ISR can optimize clinical pathways and guide timely therapeutic interventions, ultimately improving patient outcomes.

ISR was angiographically characterized by a ≥50% luminal narrowing within or bordering a previously placed stent, is a common complication after PCI (6, 7). In clinical practice, six-month follow-up restenosis rates are about 20-30% for bare-metal stents (BMS) and 5-15% for drug-eluting stents (DES) (8, 9). Data from ten year DES randomized trials reveal a target lesion revascularization incidence of around 20%, primarily due to ISR (10, 11). The pathogenesis of ISR centers on endothelial injury, triggering a cascade of neointima formation and pathological proliferation of vascular smooth muscle cell (VSMC) (9, 12, 13). In addition to procedural, anatomical, and stent-related factors, metabolic and systemic risk factors also contribute significantly to ISR development (14–16). Diabetes mellitus, particularly suboptimal glycemic control, has been well-established as a strong independent predictor of ISR (5, 9, 17, 18). However, the diagnosis of diabetes does not capture the potential continuum of glycemic exposure as a risk factor. Consequently, the precise association, including any dose-response relationship and critical threshold between glycemia and ISR risk, remains incompletely defined.

Glycated hemoglobin (HbA1c), recognized as the biomarker for assessing intermediate-term (2–3 months) glycemic control (19), holds a central role in diabetes management and cardiovascular risk stratification (20, 21). Elevated HbA1c levels drive key pathological processes of ISR, such as heightened inflammation, oxidative stress, endothelial impairment, and VSMC proliferation, resulting in neointimal hyperplasia (22, 23). In diabetic patients undergoing stent implantation, elevated HbA1c levels are independently associated with a poorer prognosis after PCI, while HbA1c variability is associated with a higher incidence of ISR (24, 25). Although previous studies have suggested an association between glycemic control and ISR in diabetic patients, it remains unclear whether relationship between HbA1c levels and ISR risk across a broader population diagnosed CAD, including non-diabetic individuals. Therefore, this study aimed to test the hypothesis that higher HbA1c levels are independently associated with an increased risk of ISR in CAD patients post-PCI.

## 2 Methods

### 2.1 Study population

This cross-sectional observational cohort study included patients with a history of prior PCI and stent implantation. We assembled retrospective cohort of 8873 CAD patients receiving stent-based PCI at the First Affiliated Hospital of Xi’an Jiaotong University, spanning January 2023 to November 2025. These data originate from the Biobank at the First Affiliated Hospital of Xi’an Jiaotong University. This study obtained ethical approval from the Ethics Committee of the First Affiliated Hospital of Xi’an Jiaotong University (Ethics Review Number: XJTU1AF2016LSL-036). This study protocol complied with the Declaration of Helsinki, and thus informed consent was waived due to its retrospective design.

The analysis included all coronary angiographies performed during this admission, regardless of the clinical indication. Indications encompassed recurrent angina, positive non-invasive stress tests, acute coronary syndrome, other evidence of myocardial ischemia, and scheduled follow-up angiographies.

The primary angiographic criterion for ISR is ≥50% luminal stenosis occurring within the stent segment or its perimeters post-PCI (6, 7). The clinical diagnosis of ISR is according to angiographic evidence of restenosis together with ischemic or ACS symptoms, typically necessitating target lesion revascularization (TLR) (26, 27).

Exclusion criteria: no coronary angiography was performed during this hospital admission (814 patients were excluded), missing crucial data for assessing ISR status, such as the absence of prior coronary stent implantation results or unclear restenosis status of the original stent (725 patients were excluded), patients lacking essential clinical and laboratory test data (248 patients were excluded). Additionally, patients whose HbA1c data were missing (789 patients were excluded). All in all, 6297 patients with CAD after PCI were recruited.

### 2.2 Data collection

The dataset for this study encompassed a range of clinical, laboratory, echocardiography, medication and coronary angiographic parameters.

Baseline data: gender, age and body mass index (BMI) were collected. Smoking and drinking history were also recorded. Collected medical history data encompassed conditions like hypertension, diabetes mellitus, heart failure, chronic kidney disease (CKD), stroke, previous myocardial infarction (MI), and the clinical diagnosis upon current admission, including stable angina, unstable angina, non−ST−elevation myocardial infarction (NSTEMI), and ST−elevation myocardial infarction (STEMI). Other baseline information included New York Heart Association functional classification (NYHA classification), including class I-IV, heart rate, systolic blood pressure (SBP) and diastolic blood pressure (DBP).

Laboratory data: all laboratory data were collected for the first time after hospital admission. High-sensitivity troponin T [hs troponin T (ng/mL)], N-terminal pro-B-type natriuretic peptide [NT-proBNP (pg/mL)], White blood cell [WBC (10^9^/L)], C-reactive protein [CRP (mg/L)], Red blood cell [RBC (10^12^/L)], Hemoglobin (g/L), Platelet (10^9^/L), Albumin (g/L), Aspartate aminotransferase [AST (U/L)], Creatinine (μmol/L), Alanine aminotransferase [ALT (U/L)], Estimated glomerular filtration rate [eGFR (ml/min/1.73 m^2^)], Glucose (mmol/L), D-dimer (mg/L), Total cholesterol [TC (mmol/L)], Triglycerides [TG (mmol/L)], Low-density lipoprotein cholesterol [LDL-C (mmol/L)] and High-density lipoprotein cholesterol [HDL-C (mmol/L)] were collected. All samples were processed and analyzed as part of routine admission laboratory testing.

Echocardiographic parameters: Left ventricular ejection fraction (LVEF) was assessed.

Coronary angiography characteristics: lesion vessels, the number and location of stents, and post-stent duration (months) were collected.

Medication: utilization of aspirin, clopidogrl/ticagrelor, angiotensin-converting enzyme inhibitor (ACEI)/angiotensin II receptor blocker (ARB), β-blockers and statins.

### 2.3 HbA1c categorization

According to the current 2023 ESC (28) and 2025 ADA guidelines (29), prediabetes is defined as HbA1c of 39–47 mmol/mol (5.7%-6.4%). So individuals were stratified into three groups: group 1, (HbA1c<5.7%), group 2, (5.7% ≤ HbA1c<6.5%), group 3, (HbA1c ≥ 6.5%). This classification strictly adheres to guidelines, categorizing the population into three clinically meaningful groups: normal glucose, prediabetes, and diabetes. Setting the upper limit of prediabetes at < 6.5% aligns seamlessly with the diagnostic threshold for diabetes (≥ 6.5%), thereby ensuring mutually exclusive, clearly defined groups for accurate comparative risk analysis.

### 2.4 Outcome assessment

ISR is defined as ≥50% luminal narrowing within the stent, occurring within or next to a previously stented coronary area, as determined by coronary angiography. ISR was assessed by quantitative coronary angiography (QCA) using dedicated software (CREALIFE, ANYTHINK Imaging Systems, China). Two experienced interventional cardiologists, each with over 10 years of experience, independently reviewed all angiograms. In cases of disagreement, a third senior interventional cardiologist adjudicated the final decision. The assessors were blinded to patients’ HbA1c levels and clinical characteristics.

**Figure 1:**
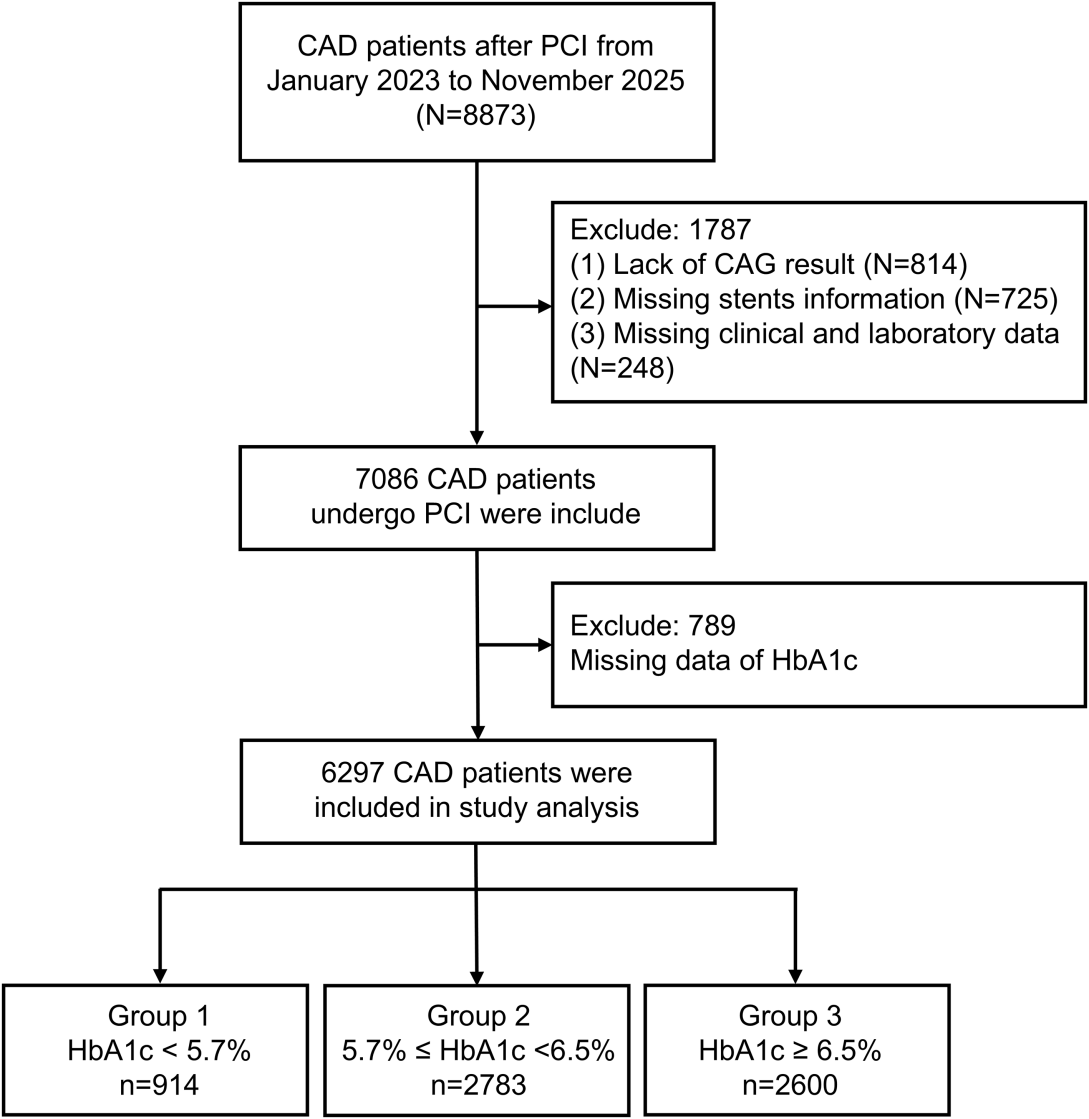
Flowchart of the study cohort. CAD, coronary artery disease; PCI, percutaneous coronary intervention; CAG, coronary angiography; HbA1c, glycated hemoglobin.

For patients with multiple lesions or stents, ISR was defined as the presence of ≥50% luminal narrowing in at least one previously stented segment. If a patient had multiple lesions meeting the ISR criterion, they were only counted once as an ISR case. **Figure 1** illustrates the patient selection and categorization process according to HbA1c levels (group 1, group 2, group 3).

### 2.5 Statistical analysis

Categorical data were presented as frequencies (percentages) and compared between groups using the chi-square test. Continuous variables were first assessed for normality using the Shapiro-Wilk test: those with a normal distribution were expressed as mean ± standard deviation (SD), while non-normally distributed variables were expressed as median with interquartile range (IQR). Comparisons between groups for all continuous variables were performed using the Kruskal-Wallis test.

The association of HbA1c with ISR risk was analyzed via univariate and multivariate logistic regression models. Using odds ratios (ORs) with 95% confidence intervals (CIs) to quantified the risk. Variables with a variance inflation factor (VIF) greater than 10 were excluded from the analysis examining the correlation between HbA1c levels and the ISR. Based on this criterion, we ultimately excluded TC from the covariate screening process. Candidate covariates were identified based on: (a) clinical relevance as established in the literature, and (b) a change-in-estimate approach, in which variables that altered the HbA1c coefficient by >10% upon addition to the model were retained. After applying these criteria, the following variables were included in the multivariable model for adjustment: gender, age, smoking history, hypertension, diabetes, heart failure, CKD, stroke, clinical diagnosis, NYHA classification, hs troponin T, CRP, WBC, RBC, platelet, LDL-C, number of lesion vessels, post-stent duration, ACEI/ARB and statins. A series of multivariable models were developed with sequential adjustment for confounding factors. Analyses were conducted with HbA1c considered both as a continuous variable and in categorical form.

The non-linear association between HbA1c levels and the ISR risk was examined using the generalized additive model (GAM). A likelihood ratio test was performed to compare the two-piecewise linear model with a standard linear logistic regression model, thereby objectively determining whether the inclusion of a threshold significantly improved model fit. Potential effect modification was assessed through stratified analyses and interaction tests based on the fully adjusted model. Statistical significance was defined as a two-sided *p* value <0.05. Analyses were conducted using Empower Stats and R software (version 4.2.0).

## 3 Results

### 3.1 Baseline characteristics

Based on HbA1c levels, individuals were divided into three categories: <5.7% (group 1), 5.7–6.4% (group 2), and ≥6.5% (group 3). As presented in **Table 1**, demographic characteristics, comprising gender, age, BMI, smoking and drinking history were comparable across groups. However, the prevalence of hypertension, diabetes and CKD was considerably higher in group 3 (*p* < 0.001 for all). Laboratory test result revealed an inverse association of hemoglobin, eGFR and HDL-C with HbA1c levels. The group 3 exhibited significant elevations in CRP, WBC, RBC and platelet count. Moreover, glucose, TC, TG and LDL-C showed a positive correlation with increasing HbA1c (*p* < 0.001 for all). For echocardiography, LVEF was inversely correlated with HbA1c levels (*p* = 0.003). SBP and heart rate increased, DBP decreased significantly with higher HbA1c levels (*p* < 0.001 for all). ACEI/ARB medications use was more frequent among individuals with elevated HbA1c levels (*p* = 0.003). Finally, the incidence of ISR progressively increased across the groups, from 15.32% in group 1 to 25.04% in group 3 (*p* < 0.001).

**Table 1:** Baseline characteristics of the study population by HbA1c categories.

Variables	HbA1c, %				*P* value
	Total	<5.7	≥5.7, <6.5	≥6.5	
	(2.70 – 14.80)	(2.70 – 5.69)	(5.70 – 6.49)	(6.50 – 14.80)	
	n = 6297	n = 914	n = 2783	n = 2600	
Demographic and clinical factors					
Male, n (%)	4941 (78.47%)	716 (78.34%)	2165 (77.79%)	2060 (79.23%)	0.438
Age, years	63.17 ± 10.30	63.00 ± 10.41	63.15 ± 10.10	63.26 ± 10.48	0.783
BMI, kg/m^2^	24.82 ± 3.14	24.73 ± 3.09	24.86 ± 3.17	24.80 ± 3.13	0.540
Smoking, n (%)	3437 (54.58%)	514 (56.67%)	1509 (54.67%)	1414 (54.87%)	0.562
Drinking, n (%)	1632 (25.92%)	241 (26.57%)	728 (26.38%)	663 (25.73%)	0.821
Hypertension, n (%)	4017 (63.79%)	518 (56.67%)	1763 (63.35%)	1736 (66.77%)	<0.001
Diabetes mellitus, n (%)	2676 (42.50%)	60 (6.56%)	439 (15.77%)	2177 (83.73%)	<0.001
Heart failure, n (%)	252 (4.00%)	36 (3.94%)	92 (3.31%)	124 (4.77%)	0.023
Chronic kidney disease, n (%)	464 (7.37%)	49 (5.36%)	160 (5.75%)	255 (9.81%)	<0.001
Stroke, n (%)	1579 (25.08%)	197 (21.55%)	710 (25.51%)	672 (25.85%)	0.028
Previous MI, n (%)	2624 (41.67%)	381 (41.68%)	1171 (42.08%)	1072 (41.23%)	0.820
Clinical Diagnosis, n (%)					0.639
Stable angina	117 (1.86%)	12 (1.31%)	55 (1.98%)	50 (1.92%)	
Unstable angina	5437 (86.34%)	801 (87.64%)	2409 (86.56%)	2227 (85.65%)	
NSTEMI	603 (9.58%)	85 (9.30%)	257 (9.23%)	261 (10.04%)	
STEMI	140 (2.22%)	16 (1.75%)	62 (2.23%)	62 (2.38%)	
NYHA Classification, n (%)					0.002
Class I	516 (8.19%)	69 (7.55%)	222 (7.98%)	225 (8.65%)	
Class II	5227 (83.01%)	774 (84.68%)	2351 (84.48%)	2102 (80.85%)	
Class III	513 (8.15%)	63 (6.89%)	198 (7.11%)	252 (9.69%)	
Class IV	41 (0.65%)	8 (0.88%)	12 (0.43%)	21 (0.81%)	
LVEF, %	61.29 ± 10.83	61.74 ± 10.28	61.70 ± 10.74	60.67 ± 11.09	0.003
First SBP, mmHg	127.39 ± 18.80	126.22 ± 17.81	126.73 ± 18.17	128.51 ± 19.72	<0.001
First DBP, mmHg	76.69 ± 10.97	77.72 ± 11.11	76.95 ± 10.97	76.06 ± 10.89	<0.001
First Heart Rate, bpm	74.31 ± 12.16	72.89 ± 11.97	73.60 ± 11.69	75.56 ± 12.60	<0.001
hs troponin T, ng/mL	0.01 (0.01-0.02)	0.01 (0.01-0.02)	0.01 (0.01-0.02)	0.01 (0.01-0.02)	<0.001
NT-proBNP, pg/mL	174.00 (71.20-527.00)	165.50 (66.03-491.50)	172.00 (70.30-532.17)	179.00 (74.70-531.00)	0.186
CRP, mg/L	10.00 (6.00-10.00)	6.00 (6.00-10.00)	10.00 (6.00-10.00)	10.00 (6.00-10.00)	0.009
WBC, 10^9^/L	6.28 ± 1.94	6.19 ± 2.04	6.26 ± 1.91	6.33 ± 1.93	0.006
RBC, 10^12^/L	4.47 ± 0.57	4.42 ± 0.61	4.45 ± 0.55	4.52 ± 0.57	<0.001
Hemoglobin, g/L	137.86 ± 17.51	138.78 ± 18.58	137.58 ± 16.73	137.83 ± 17.93	0.008
Platelet, 10^9^/L	198.37 ± 59.27	187.05 ± 57.19	199.94 ± 58.21	200.65 ± 60.67	<0.001
Albumin, g/L	42.47 ± 4.50	42.73 ± 4.37	42.55 ± 4.34	42.29 ± 4.70	0.092
AST, U/L	23.00 (19.00-29.00)	24.00 (20.00-30.00)	24.00 (20.00-29.00)	22.00 (18.00-28.00)	<0.001
ALT, U/L	25.00 (19.00-35.00)	27.00 (20.00-39.00)	25.00 (19.00-35.00)	25.00 (19.00-34.00)	<0.001
Creatinine, μmol/L	69.00 (59.00-82.00)	69.00 (60.00-81.00)	69.00 (59.00-81.00)	69.00 (58.00-84.00)	0.011
eGFR, ml/min/1.73 m^2^	89.38 ± 20.59	92.79 ± 20.77	89.94 ± 18.81	87.57 ± 22.11	<0.001
Glucose, mmol/L	6.20 (5.19-8.12)	5.31 (4.80-6.34)	5.61 (4.98-6.77)	7.84 (6.16-10.69)	<0.001
HbA1c, %	6.64 ± 1.24	5.39 ± 0.26	6.03 ± 0.22	7.72 ± 1.23	<0.001
D-dimer, mg/L	0.53 (0.42-0.70)	0.50 (0.40-0.67)	0.53 (0.43-0.71)	0.54 (0.43-0.71)	0.896
TC, mmol/L	3.27 ± 0.94	3.12 ± 0.93	3.27 ± 0.92	3.32 ± 0.95	<0.001
TG, mmol/L	1.24 (0.92-1.68)	1.14 (0.84-1.57)	1.19 (0.89-1.61)	1.32 (0.98-1.81)	<0.001
LDL-C, mmol/L	1.64 ± 0.73	1.54 ± 0.73	1.64 ± 0.71	1.68 ± 0.75	<0.001
HDL-C, mmol/L	0.93 ± 0.23	0.94 ± 0.23	0.95 ± 0.23	0.91 ± 0.22	<0.001
Number of lesion vessels, n (%)					<0.001
1	526 (8.35%)	93 (10.18%)	289 (10.38%)	144 (5.54%)	
2	1231 (19.55%)	220 (24.07%)	578 (20.77%)	433 (16.65%)	
3	4540 (72.10%)	601 (65.75%)	1916 (68.85%)	2023 (77.81%)	
LM lesion, n (%)	428 (6.80%)	63 (6.89%)	208 (7.47%)	157 (6.04%)	0.111
LAD lesion, n (%)	3261 (51.79%)	457 (50.00%)	1475 (53.00%)	1329 (51.12%)	0.194
LCX lesion, n (%)	1426 (22.65%)	181 (19.80%)	633 (22.75%)	612 (23.54%)	0.067
RCA lesion, n (%)	2745 (43.59%)	409 (44.75%)	1148 (41.25%)	1188 (45.69%)	0.003
Post-stent duration, months	12.00 (2.00-60.00)	8.00 (1.00-60.00)	12.00 (2.00-60.00)	15.00 (2.00-72.00)	0.015
Aspirin, n (%)	3127 (49.66%)	458 (50.11%)	1373 (49.34%)	1296 (49.85%)	0.893
Clopidogrl/Ticagrelor, n (%)	2664 (42.31%)	396 (43.33%)	1198 (43.05%)	1070 (41.15%)	0.297
ACEI/ARB, n (%)	2173 (34.51%)	283 (30.96%)	936 (33.63%)	954 (36.69%)	0.003
β-blockers, n (%)	1538 (24.42%)	216 (23.63%)	658 (23.64%)	664 (25.54%)	0.226
Statins, n (%)	2798 (44.43%)	423 (46.28%)	1225 (44.02%)	1150 (44.23%)	0.472
ISR, n (%)	1305 (20.72%)	140 (15.32%)	514 (18.47%)	651 (25.04%)	<0.001

Data are shown as mean ± SD, median (IQR), or numbers (percentages).

**Supplementary Table 1** indicated that ISR patients typically had an older age profile and a history of smoking. They exhibited a notably greater prevalence of hypertension, diabetes, heart failure or CKD. In terms of clinical diagnosis, patients with ISR had a higher proportion of being diagnosed with NSTEMI and STEMI. Moreover, they had a higher proportion of being classified as NYHA Class III and IV. Patients with ISR exhibited elevated levels of CRP, WBC, platelets, creatinine, glucose, HbA1c, D-dimer, TC, TG and LDL-C, alongside reduced levels of RBC, hemoglobin, albumin and eGFR. Patients with ISR exhibited increased SBP and heart rate, decreased DBP. In terms of medication utilization, a lower proportion of ISR patients were prescribed statins compared to non-ISR patients.

### 3.2 Univariate logistic regression analysis for ISR

**Supplementary Table 2** indicated that age, smoking history, hypertension, diabetes, heart failure and CKD were associated with an increased risk of ISR. Elevated levels of HbA1c, glucose, hs troponin T, CRP, TC, TG and LDL-C were linked to a higher ISR risk, whereas elevated RBC, hemoglobin, albumin and eGFR levels were correlated with lower ISR risk. Echocardiographic analyses indicated that a higher heart rate was associated with an increased ISR risk, whereas a higher LVEF was associated with a decreased ISR risk. In addition, longer stent implantation time was linked to a higher ISR risk. The use of statins was linked to a decreased risk of ISR.

### 3.3 Analysis of multivariate logistic regression models

**Table 2** showed a consistent association between elevated HbA1c levels and increased ISR probability. We evaluated the robustness of this association by developing four models with increasing levels of adjustment. Model 1 was constructed to evaluate the unadjusted association between HbA1c levels and ISR. Model 2 accounted for basic demographic factors, namely gender and age. Model 3 was further adjusted for key covariates, including smoking history, significant comorbidities (hypertension, diabetes, heart failure, CKD and stroke), as well as clinical diagnosis and NYHA classification. Model 4, our fully adjusted model, incorporated all variables from Model 3, as well as laboratory parameters (hs troponin T, CRP, WBC, RBC, platelet, LDL-C), number of lesion vessels, post-stent duration and medication history (ACEI/ARB and statins). Elevated HbA1c correlated with increased risk of ISR, and this association remained robust across model 1 to model 4.

**Table 2:** Multivariable logistic regression models for ISR risk based on HbA1c levels.

	Model 1	Model 2	Model 3	Model 4
Exposure	OR (95%CI) *P*	OR (95%CI) *P*	OR (95%CI) *P*	OR (95%CI) *P*
HbA1c, %	1.20 (1.14, 1.25) <0.0001	1.20 (1.14, 1.25) <0.0001	1.18 (1.11, 1.26) <0.0001	1.18 (1.09, 1.27) <0.0001
HbA1c, % group				
< 5.7	Reference	Reference	Reference	Reference
≥ 5.7, < 6.5	1.25 (1.02, 1.54) 0.0305	1.25 (1.02, 1.54) 0.0305	1.23 (1.01, 1.50) 0.0390	1.11 (0.88, 1.40) 0.3844
≥ 6.5	1.85 (1.51, 2.26) <0.0001	1.84 (1.51, 2.26) <0.0001	1.65 (1.28, 2.11) <0.0001	1.70 (1.26, 2.29) 0.0005
*P* for trend	1.40 (1.27, 1.53) <0.0001	1.39 (1.27, 1.53) <0.0001	1.28 (1.13, 1.45) <0.0001	1.30 (1.12, 1.52) 0.0006

Model 1: unadjusted.

Model 2: adjusted for demographics (gender and age).

Model 3: adjusted for gender, age, smoking history, hypertension, diabetes, heart failure, CKD, stroke, clinical diagnosis and NYHA classification.

Model 4: fully adjusted model: includes all covariates in Model 3 plus laboratory results (hs troponin T, CRP, WBC, RBC, platelet, LDL-C), number of lesion vessels, post-stent duration, ACEI/ARB and statins.

In the categorical analysis, patients with elevated HbA1c (≥6.5%) showed a significantly increased likelihood of ISR. The association persisted strongly in Model 4 after comprehensive adjustments, with an OR of 1.70 (*p* = 0.0005). The trend test for HbA1c levels demonstrated statistical significance across all models, supporting a dose-response relationship between higher HbA1c levels and increased ISR risk.

### 3.4 Association of HbA1c levels with ISR risk in coronary artery disease patients

**Figure 2:**
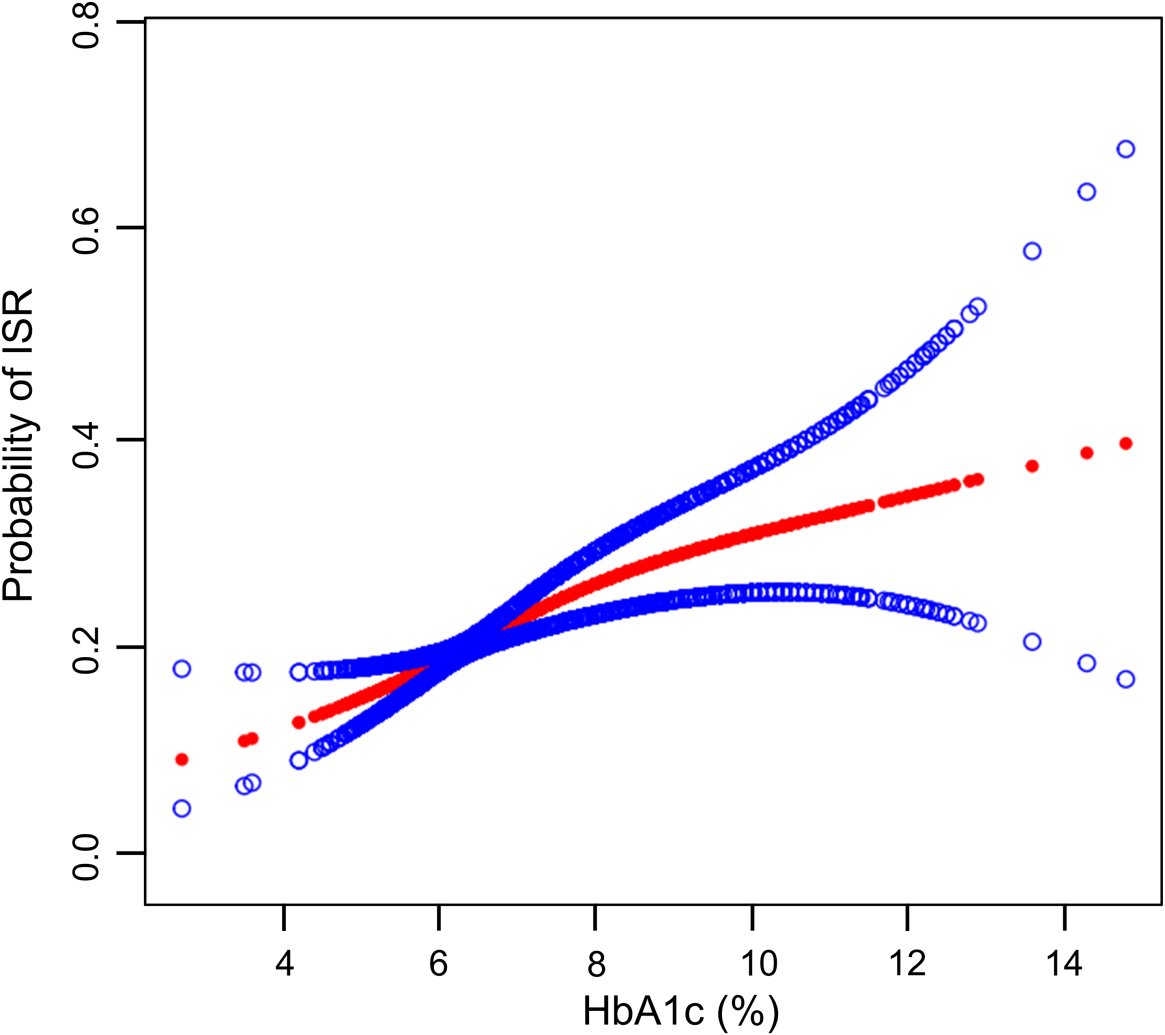
Associations between the HbA1c levels and ISR. After full adjustment for all covariates in Model 4 (including gender, age, smoking history, hypertension, diabetes, heart failure, CKD, stroke, clinical diagnosis, NYHA classification, hs troponin T, CRP, WBC, RBC, platelet, LDL-C, number of lesion vessels, post-stent duration, ACEI/ARB and statins), the relationship between HbA1c levels and ISR risk showed a non-linear pattern. The red line shows the estimated values, with the blue line indicating the associated 95%CIs.

Using GAM analysis (Model 4, fully adjusted), we examined the hypothesized association between HbA1c levels and ISR risk. This model encompassed a comprehensive set of covariates, including gender, age, smoking history, hypertension, diabetes, heart failure, CKD, stroke, clinical diagnosis, NYHA classification, hs troponin T, CRP, WBC, RBC, platelet, LDL-C, number of lesion vessels, post-stent duration, ACEI/ARB and statins. **Figure 2** illustrated a non-linear association between HbA1c levels and the likelihood of ISR in individuals with CAD after PCI. The probability of ISR rose markedly with higher HbA1c levels. The adjustment for multiple confounders in the model underscored the robust link between high HbA1c levels and ISR risk in CAD patients after PCI.

Threshold effect analysis showed a significant non-linear association between HbA1c levels and ISR risk (**Table 3**). Specifically, in its linear segment (Model I), every one-unit increment in HbA1c level corresponded to an 18% elevation in ISR risk (OR: 1.18, *p* < 0.0001). Similarly, each SD increase corresponded to a 23% increased risk (OR: 1.23, *p* < 0.0001). In Model II (non-linear analysis), a turning point (K) was observed at an HbA1c of 7.8%. Below this point, the risk of ISR increased significantly with rising HbA1c (per-unit OR: 1.37, *p* < 0.0001; per-SD OR: 1.49, *p* < 0.0001). However, beyond this threshold, this association weakened substantially and ceased to be statistically significant. The log-likelihood ratio test (LRT) showed a superior fit for the non-linear model (Model II) over the linear model (Model I) (p= 0.024).

**Table 3:** Threshold effect analysis of HbA1c on ISR risk.

	HbA1c per-unit increase		HbA1c per-SD increase	
Models	OR (95%CI)	*P* value	OR (95%CI)	*P* value
Model I.				
One line effect	1.18 (1.09, 1.27)	<0.0001	1.23 (1.11, 1.35)	<0.0001
Model II				
Turning point (K)	7.8		0.93	
HbA1c < K	1.37 (1.17, 1.60)	<0.0001	1.49 (1.22, 1.80)	<0.0001
HbA1c > K	1.04 (0.91, 1.19)	0.5485	1.05 (0.89, 1.24)	0.5484
*P* value for LRT test		0.024		0.024

Model I, linear analysis; Model II, non-linear analysis. Adjusted for: gender, age, smoking history, hypertension, diabetes, heart failure, CKD, stroke, clinical diagnosis, NYHA classification, hs troponin T, CRP, WBC, RBC, platelet, LDL-C, number of lesion vessels, post-stent duration, ACEI/ARB and statins). Model II differs significantly from Model I by the logarithm likelihood ratio test (LRT) by *p* < 0.05.

### 3.5 Analysis of interaction effects by stratified characteristics

**Figure 3:**
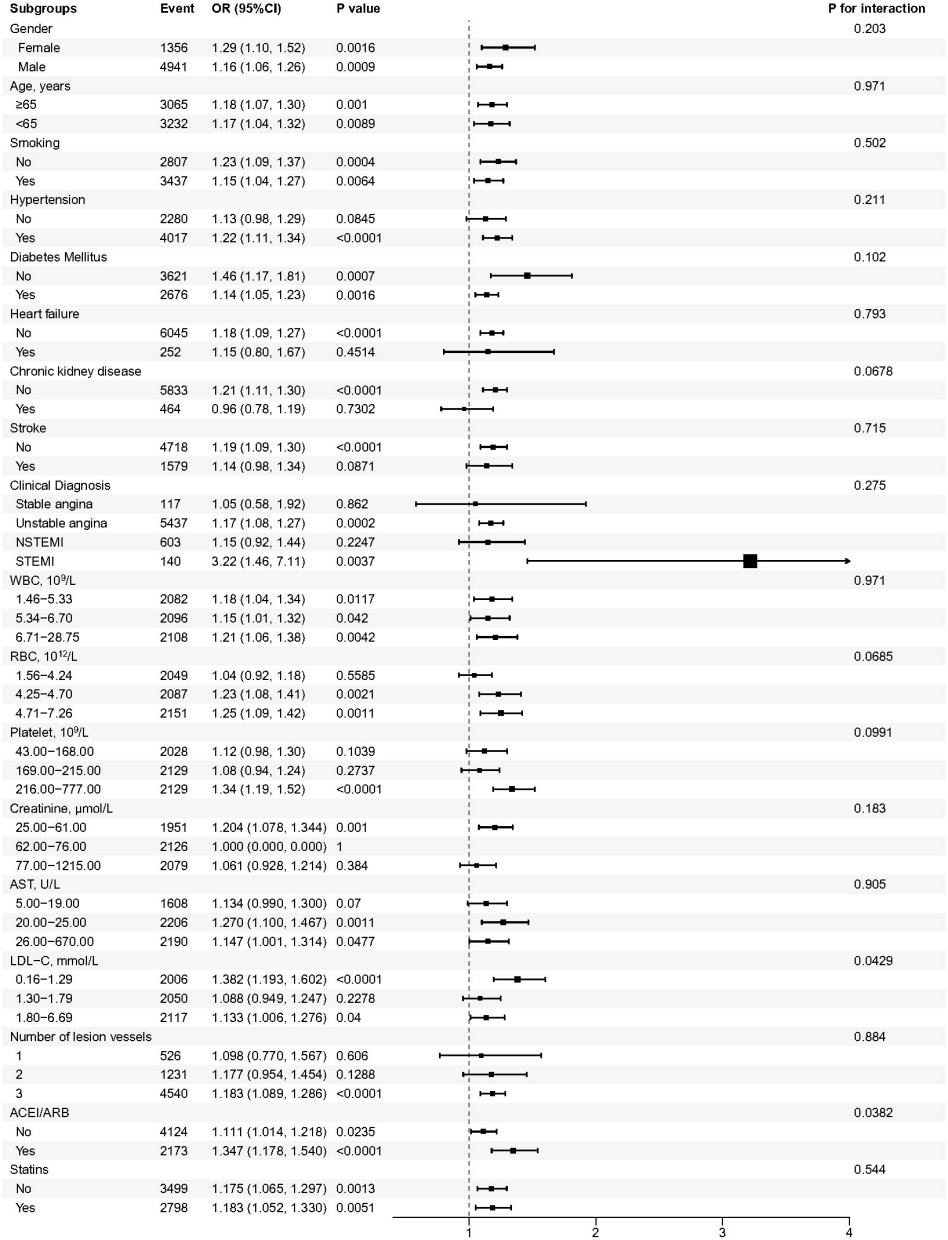
Stratified subgroup analysis by HbA1c levels for ISR in CAD patients post-PCI. The adjustment accounted for all variables specified in the fully adjusted model, with the exception of the stratification element. The cutoffs for WBC, RBC, platelet, creatinine, AST, and LDL-C were defined based on the tertiles of their levels in the study population.

To evaluate the consistency of the HbA1c-ISR association, subgroup analyses were conducted. As shown in **Figure 3**, the relationships between HbA1c and ISR were heterogeneous across different subgroups. The link between high HbA1c levels and ISR risk was not consistently evident across subgroups divided by hypertension, heart failure, CKD or stroke. Notably, significant interactions were observed for LDL-C (p for interaction = 0.0429) and ACEI/ARB use (p for interaction = 0.0382). Specifically, this relationship was more pronounced in patients with lower LDL-C levels (defined as <1.29 mmol/L) and in those who were taking ACEI/ARB medications.

The analysis found no significant interactions in other subgroups based on gender, age, smoking history, hypertension, diabetes, heart failure, CKD, stroke, clinical diagnosis, laboratory test result including WBC, RBC, platelet, creatinine, AST or medication use (statins), indicating that the association between HbA1c and ISR risk remains consistent across these clinical and treatment variables.

## 4 Discussion

**Figure 4:**
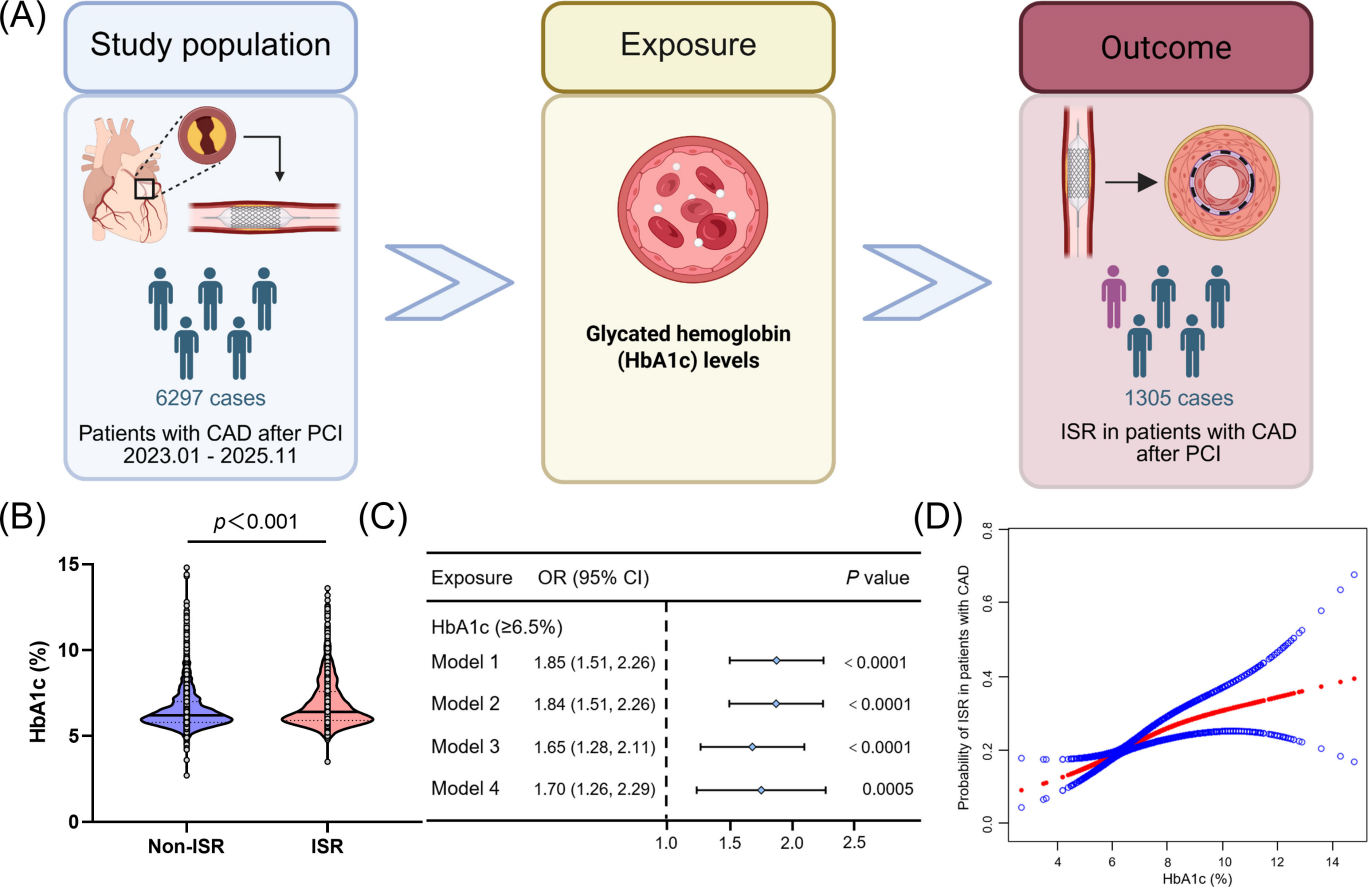
Graphical abstract. **(A)** Among 6297 CAD patients following PCI in this retrospective cohort study, 1305 patients (20.7%) developed ISR. **(B)** Patients who developed ISR had significantly higher HbA1c levels. **(C)** Across different models, HbA1c ≥6.5% was independently associated with ISR in patients with CAD after PCI. **(D)** HbA1c levels exhibited a non-linear association with the incidence of ISR.

ISR was a frequent clinical complication following PCI. As PCI techniques advance rapidly and their indications expand, the incidence of ISR had also increased (30). As shown in **Figure 4**, our study yielded three principal findings: First, participants with ISR exhibited significantly higher HbA1c levels than those without. Second, elevated HbA1c was associated with an increased risk of ISR. Patients with HbA1c ≥ 6.5% had a 70% higher risk of ISR. Finally, the relationship was non-linear with a threshold effect, with a particularly strong association observed at HbA1c levels below 7.8%.

Beyond stent-related characteristics (such as number, length, and diameter) and genetic predisposition, poor lifestyle habits and metabolic abnormalities were also major factors influencing the occurrence of ISR (31–33). Our study identified higher HbA1c levels as an independent associated factor for ISR, consistent with other studies. Fujita et al. found uncontrolled glycemia at follow-up significantly elevated TLR risk in comparison to non-diabetic patients. The study emphasized the significance of optimal glycemic control in minimizing late restenosis for diabetic patients post-PCI (34). Santos-Pardo et al. showed that poor glycemic control, defined by higher HbA1c levels (>7.1%), were significantly linked to an elevated risk of stent failure. This investigation underscored the significance of effective glycemic management in minimizing stent failure risk for diabetic patients undergoing PCI (35). While the aforementioned studies exclusively enrolled diabetic populations, the present study extended this scope by including non-diabetic patients as well. By treating HbA1c as a continuous variable and identifying a specific risk threshold, our findings broaden the applicable population and provide a more precise reference for risk stratification based on HbA1c levels.

By integrating GAM with threshold effect analysis, this study provided a robust framework for identifying and validating the non-linear association of HbA1c with ISR risk. We applied a two-piecewise logistic regression approach to detect and rigorously estimate the threshold, identifying 7.8% as the optimal cut-point by maximizing the likelihood ratio statistic compared to a traditional single-line logistic model. Below this threshold, the association with ISR risk becomes markedly stronger. Our findings indicated that for patients undergoing PCI with HbA1c levels not exceeding 7.8%, the observed association between HbA1c and ISR risk was markedly stronger. This distinct pattern warrants particular attention in patients with elevated HbA1c, highlighting them as a population requiring closer monitoring. It is important to note that our study cohort comprised patients with established CAD who had previously undergone PCI, a population characterized by a high burden of metabolic comorbidities (42.50% with diagnosed diabetes; mean HbA1c 6.64% ± 1.24%). In this context, 14.4% of the entire cohort (907 patients) had HbA1c levels above 7.8%, indicating that this threshold corresponds to a clinically meaningful subgroup rather than an extreme tail of the distribution. The observed inflection points at 7.8% should be interpreted cautiously as an exploratory finding; prospective studies are needed to confirm whether this threshold represents a clinically meaningful transition in risk.

The association between HbA1c and ISR was consistent across subgroups including gender, age, comorbidities, laboratory parameters and statin therapy, suggesting that elevated HbA1c is a robust predictor of ISR, independent of these clinical characteristics. However, the association between HbA1c levels and ISR risk was found to be dependent on two factors: use of ACEI/ARB medications and achieving a lower LDL-C level. Interestingly, Ujiie et al. observed that ACEI may actually increase the risk of ISR when combined with aspirin and cilostazol, suggesting that ACEI might promote intimal proliferation following stent implantation (36). Carriers of the ACE gene deletion (DD) polymorphism exhibited an increased incidence of ISR in a *post hoc* analysis that stratified patients by ACEI/ARB treatment, suggesting genetic risk factor for restenosis following coronary stenting (37–39). ACEI may elevate ISR incidence through bradykinin accumulation, which triggers inflammation and smooth muscle cell proliferation via bradykinin B2 receptors and the MAPK pathway (40). In addition, patients receiving ACEI/ARB therapy often present with more complex clinical profiles, including conditions such as hypertension, heart failure, and diabetic nephropathy. This complexity is an indicator of a higher underlying risk, which is independently associated with an increased probability of ISR (41, 42). Our findings highlight that for patients prescribed ACEI/ARB, the relationship between glycemic control and ISR risk may be more complex. This observation underscores the need for heightened awareness and further research to determine the optimal monitoring and management strategies for this specific subgroup.

For the aspect of LDL-C, low levels of LDL-C effectively suppress lipid core formation and inflammation-driven ISR. Consequently, other pathological pathways driven by hyperglycemia become relatively more prominent, emerging as the primary drivers of ISR. Patients with elevated HbA1c who have received coronary stents warrant close monitoring as a high-risk group for ISR. Optimal glycemic control should be prioritized for all patients, irrespective of their lipid levels. As part of an integrated approach to cardiovascular risk reduction, glucose-lowering agents with established cardio-renal benefits, including GLP-1 receptor agonists and SGLT2 inhibitors, are strongly recommended (43, 44).

The study is subject to several limitations. First, its retrospective design precludes the establishment of causality. Second, the findings may have limited generalizability due to the single-center data. Third, our dataset lacked information on procedural and pharmacological factors, including stent dimensions, lesion complexity, post-dilatation, intravascular imaging use, and medication adherence or intensity. Therefore, despite multivariable adjustment for known confounders, the possibility of residual confounding due to these unmeasured variables remains. Fourth, a potential selection bias was introduced by the necessary exclusion of post-PCI CAD patients who did not undergo angiography and patients with missing HbA1c data, we compared baseline characteristics between included and excluded individuals (**Supplementary Table 3**). The overall direction and magnitude of selection bias are difficult to determine. This inherent uncertainty should be considered when interpreting the generalizability of our results. Finally, the statistical power for the subgroup with HbA1c levels above the identified 7.8% threshold was limited, as it comprised only 963 patients. This constrained our ability to precisely characterize the association pattern in this higher glycemic range and may affect the stability of the threshold estimate itself.

## 5 Conclusions

In summary, this study revealed a strong non-linear link between high HbA1c levels and ISR occurrence in CAD patients post-PCI. Identifying a 7.8% threshold provided new insights into the association between HbA1c levels and ISR. These findings highlight the potential value of HbA1c in post-PCI risk stratification, though further prospective studies are needed to validate its clinical utility.

## Data Availability

The original contributions presented in the study are included in the article/**Supplementary Material**. Further inquiries can be directed to the corresponding author.
