# Supplementary material for: The association between glycated hemoglobin levels and in-stent restenosis following percutaneous coronary intervention in coronary artery disease patients
Source: Front Endocrinol (Lausanne). 2026 Apr 23;17:1793093. doi: 10.3389/fendo.2026.1793093 (PMC13149118; doi:10.3389/fendo.2026.1793093)
Supplement: Supplementary file 1 [file Table1.docx]

**Table S1.** Baseline characteristics stratified by ISR presence.

| Group | Total | Non-ISR | ISR |  |
| --- | --- | --- | --- | --- |
|  | n = 6297 | n = 4992 | n = 1305 | *P* value |
| Demographic and clinical factors | | | | |
| Male, n (%) | 4941 (78.47%) | 3901 (78.15%) | 1040 (79.69%) | 0.226 |
| Age, years | 63.17 ± 10.30 | 62.57 ± 10.33 | 65.47 ± 9.86 | <0.001 |
| BMI, kg/m^2^ | 24.82 ± 3.14 | 24.83 ± 3.14 | 24.78 ± 3.15 | 0.630 |
| Smoking, n (%) | 3437 (54.58%) | 2687 (54.33%) | 750 (57.78%) | 0.026 |
| Drinking, n (%) | 1632 (25.92%) | 1286 (26.00%) | 346 (26.66%) | 0.632 |
| Hypertension, n (%) | 4017 (63.79%) | 3075 (61.60%) | 942 (72.18%) | <0.001 |
| Diabetes mellitus, n (%) | 2676 (42.50%) | 2010 (40.26%) | 666 (51.03%) | <0.001 |
| Heart failure, n (%) | 252 (4.00%) | 178 (3.57%) | 74 (5.67%) | <0.001 |
| Chronic kidney disease, n (%) | 464 (7.37%) | 134 (2.68%) | 330 (25.29%) | <0.001 |
| Stroke, n (%) | 1579 (25.08%) | 1241 (24.86%) | 338 (25.90%) | 0.440 |
| Previous MI, n (%) | 2624 (41.67%) | 2051 (41.09%) | 573 (43.91%) | 0.066 |
| Clinical Diagnosis, n (%) | | | | <0.001 |
| Stable angina | 117 (1.86%) | 84 (1.68%) | 33 (2.53%) |  |
| Unstable angina | 5437 (86.34%) | 4483 (89.80%) | 954 (73.10%) |  |
| NSTEMI | 603 (9.58%) | 351 (7.03%) | 252 (19.31%) |  |
| STEMI | 140 (2.22%) | 74 (1.48%) | 66 (5.06%) |  |
| NYHA Classification, n (%) | | | | <0.001 |
| Class I | 516 (8.19%) | 406 (8.13%) | 110 (8.43%) |  |
| Class II | 5227 (83.01%) | 4188 (83.89%) | 1039 (79.62%) |  |
| Class III | 513 (8.15%) | 371 (7.43%) | 142 (10.88%) |  |
| Class IV | 41 (0.65%) | 27 (0.54%) | 14 (1.07%) |  |
| LVEF, % | 61.29 ± 10.83 | 61.45 ± 10.67 | 60.67 ± 11.39 | 0.209 |
| First SBP, mmHg | 127.39 ± 18.80 | 127.17 ± 18.80 | 128.23 ± 18.78 | 0.028 |
| First DBP, mmHg | 76.69 ± 10.97 | 76.89 ± 10.87 | 75.96 ± 11.33 | 0.007 |
| First Heart Rate, bpm | 74.31 ± 12.16 | 74.02 ± 12.01 | 75.42 ± 12.66 | <0.001 |
| hs troponin T, ng/mL | 0.01 (0.01-0.02) | 0.01 (0.01-0.02) | 0.01 (0.01-0.03) | <0.001 |
| NT-proBNP, pg/mL | 174.00 (71.20-527.00) | 174.80 (69.70-529.15) | 173.00 (77.80-502.00) | 0.606 |
| CRP, mg/L | 10.00 (6.00-10.00) | 7.40 (6.00-10.00) | 10.00 (6.00-10.00) | <0.001 |
| WBC, 10^9^/L | 6.28 ± 1.94 | 6.25 ± 1.93 | 6.38 ± 1.94 | 0.032 |
| RBC, 10^12^/L | 4.47 ± 0.57 | 4.51 ± 0.54 | 4.31 ± 0.62 | <0.001 |
| Hemoglobin, g/L | 137.86 ± 17.51 | 139.40 ± 16.70 | 131.91 ± 19.22 | <0.001 |
| Platelet, 10^9^/L | 198.37 ± 59.27 | 197.18 ± 57.99 | 202.94 ± 63.76 | 0.002 |
| Albumin, g/L | 42.47 ± 4.50 | 42.66 ± 4.35 | 41.64 ± 5.00 | <0.001 |
| AST, U/L | 23.00 (19.00-29.00) | 23.00 (19.00-29.00) | 23.00 (19.00-29.00) | 0.265 |
| ALT, U/L | 25.00 (19.00-35.00) | 26.00 (19.00-36.00) | 24.00 (18.00-33.00) | <0.001 |
| Creatinine, μmol/L | 69.00 (59.00-82.00) | 68.00 (60.00-78.00) | 101.00 (46.00-119.25) | <0.001 |
| eGFR, ml/min/1.73 m^2^ | 89.38 ± 20.59 | 92.46 ± 15.41 | 77.39 ± 31.11 | <0.001 |
| Glucose, mmol/L | 6.20 (5.19-8.12) | 6.12 (5.16-7.96) | 6.55 (5.33-8.94) | <0.001 |
| HbA1c, % | 6.64 ± 1.24 | 6.57 ± 1.19 | 6.88 ± 1.36 | <0.001 |
| D-dimer, mg/L | 0.53 (0.42-0.70) | 0.53 (0.41-0.68) | 0.58 (0.46-0.82) | <0.001 |
| TC, mmol/L | 3.27 ± 0.94 | 3.25 ± 0.93 | 3.36 ± 0.95 | <0.001 |
| TG, mmol/L | 1.24 (0.92-1.68) | 1.22 (0.91-1.66) | 1.30 (0.93-1.78) | 0.016 |
| LDL-C, mmol/L | 1.64 ± 0.73 | 1.62 ± 0.72 | 1.70 ± 0.74 | <0.001 |
| HDL-C, mmol/L | 0.93 ± 0.23 | 0.93 ± 0.22 | 0.94 ± 0.24 | 0.344 |
| Number of lesion vessels, n (%) | | | | 0.141 |
| 1 | 526 (8.35%) | 428 (8.57%) | 98 (7.51%) |  |
| 2 | 1231 (19.55%) | 993 (19.89%) | 238 (18.24%) |  |
| 3 | 4540 (72.10%) | 3571 (71.53%) | 969 (74.25%) |  |
| LM lesion, n (%) | 428 (6.80%) | 333 (6.67%) | 95 (7.28%) | 0.436 |
| LAD lesion, n (%) | 3261 (51.79%) | 2580 (51.68%) | 681 (52.18%) | 0.747 |
| LCX lesion, n (%) | 1426 (22.65%) | 1132 (22.68%) | 294 (22.53%) | 0.91 |
| RCA lesion, n (%) | 2745 (43.59%) | 2180 (43.67%) | 565 (43.30%) | 0.808 |
| Post-stent duration, months | 12.00 (2.00-60.00) | 6.00 (1.00-48.00) | 60.00 (24.00-120.00) | <0.001 |
| Aspirin, n (%) | 3127 (49.66%) | 2506 (50.20%) | 621 (47.59%) | 0.093 |
| Clopidogrl/Ticagrelor, n (%) | 2664 (42.31%) | 2139 (42.85%) | 525 (40.23%) | 0.088 |
| ACEI/ARB, n (%) | 2173 (34.51%) | 1720 (34.46%) | 453 (34.71%) | 0.862 |
| β-blockers, n (%) | 1538 (24.42%) | 1228 (24.60%) | 310 (23.75%) | 0.527 |
| Statins, n (%) | 2798 (44.43%) | 2257 (45.21%) | 541 (41.46%) | 0.015 |

Data are shown as mean ± SD, median (IQR), or numbers (percentages).
