# Supplementary material for: The association between glycated hemoglobin levels and in-stent restenosis following percutaneous coronary intervention in coronary artery disease patients
Source: Front Endocrinol (Lausanne). 2026 Apr 23;17:1793093. doi: 10.3389/fendo.2026.1793093 (PMC13149118; doi:10.3389/fendo.2026.1793093)
Supplement: Supplementary file 2 [file Table2.docx]

**Table S2.** Univariate logistic regression analysis for ISR.

| Exposure | Statistics | OR (95% CI) | *P* value |
| --- | --- | --- | --- |
| Male, n (%) | 4941 (78.47%) | 1.10 (0.94, 1.28) | 0.2258 |
| Age, years | 63.17 ± 10.30 | 1.03 (1.02, 1.04) | <0.0001 |
| BMI, kg/m^2^ | 24.82 ± 3.14 | 1.00 (0.98, 1.02) | 0.6301 |
| Smoking, n (%) | 3437 (55.04%) | 1.15 (1.02, 1.30) | 0.026 |
| Drinking, n (%) | 1632 (26.14%) | 1.03 (0.90, 1.19) | 0.6324 |
| Hypertension, n (%) | 4017 (63.79%) | 1.62 (1.42, 1.85) | <0.0001 |
| Diabetes mellitus, n (%) | 2676 (42.50%) | 1.55 (1.37, 1.75) | <0.0001 |
| Heart failure, n (%) | 252 (4.00%) | 1.63 (1.23, 2.15) | 0.0006 |
| Chronic kidney disease, n (%) | 464 (7.37%) | 12.27 (9.92, 15.17) | <0.0001 |
| Stroke, n (%) | 1579 (25.08%) | 1.06 (0.92, 1.21) | 0.44 |
| Previous MI, n (%) | 2624 (41.67%) | 1.12 (0.99, 1.27) | 0.0657 |
| Clinical Diagnosis, n (%) | | | |
| Stable angina | 117 (1.86%) | 1 |  |
| Unstable angina | 5437 (86.34%) | 0.54 (0.36, 0.82) | 0.0033 |
| NSTEMI | 603 (9.58%) | 1.83 (1.18, 2.82) | 0.0065 |
| STEMI | 140 (2.22%) | 2.27 (1.35, 3.83) | 0.0021 |
| NYHA Classification, n (%) | | | |
| Class I | 516 (8.19%) | 1 |  |
| Class II | 5227 (83.01%) | 0.92 (0.73, 1.14) | 0.4354 |
| Class III | 513 (8.15%) | 1.41 (1.06, 1.88) | 0.0179 |
| Class IV | 41 (0.65%) | 1.91 (0.97, 3.77) | 0.061 |
| LVEF, % | 61.29 ± 10.83 | 0.99 (0.99, 1.00) | 0.0359 |
| First SBP, mmHg | 127.39 ± 18.80 | 1.00 (1.00, 1.01) | 0.0698 |
| First DBP, mmHg | 76.69 ± 10.97 | 0.99 (0.99, 1.00) | 0.0067 |
| First Heart Rate, bpm | 74.31 ± 12.16 | 1.01 (1.00, 1.01) | 0.0002 |
| hs troponin T, ng/mL | 0.09 ± 0.48 | 1.27 (1.13, 1.43) | <0.0001 |
| NT-proBNP, pg/mL | 884.43 ± 3151.34 | 1.00 (1.00, 1.00) | 0.6061 |
| CRP, mg/L | 9.20 ± 8.73 | 1.01 (1.01, 1.02) | 0.0004 |
| WBC, 10^9^/L | 6.28 ± 1.94 | 1.03 (1.00, 1.07) | 0.0321 |
| RBC, 10^12^/L | 4.47 ± 0.57 | 0.54 (0.48, 0.60) | <0.0001 |
| Hemoglobin, g/L | 137.86 ± 17.51 | 0.98 (0.97, 0.98) | <0.0001 |
| Platelet, 10^9^/L | 198.37 ± 59.27 | 1.00 (1.00, 1.00) | 0.0019 |
| Albumin, g/L | 42.47 ± 4.50 | 0.95 (0.94, 0.97) | <0.0001 |
| AST, U/L | 27.99 ± 28.64 | 1.00 (1.00, 1.00) | 0.0416 |
| ALT, U/L | 31.14 ± 26.91 | 1.00 (1.00, 1.00) | 0.1612 |
| Creatinine, μmol/L | 81.46 ± 79.69 | 1.00 (1.00, 1.00) | <0.0001 |
| eGFR, ml/min/1.73 m^2^ | 89.38 ± 20.59 | 0.97 (0.97, 0.97) | <0.0001 |
| Glucose, mmol/L | 7.22 ± 3.14 | 1.07 (1.05, 1.09) | <0.0001 |
| HbA1c, % | 6.64 ± 1.24 | 1.20 (1.15, 1.26) | <0.0001 |
| D-dimer, mg/L | 10.18 ± 141.35 | 1.00 (1.00, 1.00) | 0.8499 |
| TC, mmol/L | 3.27 ± 0.94 | 1.13 (1.06, 1.20) | 0.0003 |
| TG, mmol/L | 1.45 ± 1.00 | 1.07 (1.01, 1.13) | 0.017 |
| LDL-C, mmol/L | 1.64 ± 0.73 | 1.15 (1.06, 1.25) | 0.0009 |
| HDL-C, mmol/L | 0.93 ± 0.23 | 1.14 (0.87, 1.49) | 0.3439 |
| Number of lesion vessels, n (%) | | | |
| 1 | 526 (8.35%) | 1 |  |
| 2 | 1231 (19.55%) | 1.05 (0.81, 1.36) | 0.7316 |
| 3 | 4540 (72.10%) | 1.19 (0.94, 1.49) | 0.1491 |
| LM lesion, n (%) | 428 (6.80%) | 1.10 (0.87, 1.39) | 0.4365 |
| LAD lesion, n (%) | 3261 (51.79%) | 1.02 (0.90, 1.15) | 0.747 |
| LCX lesion, n (%) | 1426 (22.65%) | 0.99 (0.86, 1.15) | 0.9097 |
| RCA lesion, n (%) | 2745 (43.59%) | 0.98 (0.87, 1.11) | 0.8079 |
| Post-stent duration, months | 40.19 ± 52.41 | 1.01 (1.01, 1.02) | <0.0001 |
| Aspirin, n (%) | 3127 (49.66%) | 0.90 (0.80, 1.02) | 0.0927 |
| Clopidogrl/Ticagrelor, n (%) | 2664 (42.31%) | 0.90 (0.79, 1.02) | 0.0883 |
| ACEI/ARB, n (%) | 2173 (34.51%) | 1.01 (0.89, 1.15) | 0.8617 |
| β-blockers, n (%) | 1538 (24.42%) | 0.95 (0.83, 1.10) | 0.5272 |
| Statins, n (%) | 2798 (44.43%) | 0.86 (0.76, 0.97) | 0.0151 |

Abbreviations: OR odds ratio; CIs confidence intervals.
