# Supplementary material for: The association between glycated hemoglobin levels and in-stent restenosis following percutaneous coronary intervention in coronary artery disease patients
Source: Front Endocrinol (Lausanne). 2026 Apr 23;17:1793093. doi: 10.3389/fendo.2026.1793093 (PMC13149118; doi:10.3389/fendo.2026.1793093)
Supplement: Supplementary file 3 [file Table3.docx]

**Table S3.** Baseline characteristics of included and excluded populations.

| Group | Exclusion | Inclusion | P value |
| --- | --- | --- | --- |
|  | n = 2576 | n = 6297 |  |
| Male, n (%) | 2014 (78.18%) | 4941 (78.47%) | 0.769 |
| Age, years | 63.28 ± 10.36 | 63.17 ± 10.30 | 0.655 |
| BMI, kg/m^2^ | 24.90 ± 3.22 | 24.82 ± 3.14 | 0.344 |
| Smoking, n (%) | 1090 (49.91%) | 3437 (55.04%) | <0.001 |
| Drinking, n (%) | 514 (23.51%) | 1632 (26.14%) | 0.015 |
| Hypertension, n (%) | 1848 (71.74%) | 4017 (63.79%) | <0.001 |
| Diabetes mellitus, n (%) | 1593 (61.84%) | 2676 (42.50%) | <0.001 |
| Heart failure, n (%) | 85 (3.55%) | 252 (4.00%) | 0.335 |
| Chronic kidney disease, n (%) | 126 (5.27%) | 464 (7.37%) | <0.001 |
| Stroke, n (%) | 555 (23.31%) | 1579 (25.08%) | 0.088 |
| Clinical Diagnosis, n (%) | | | <0.001 |
| Stable angina | 49 (2.05%) | 117 (1.86%) |  |
| Unstable angina | 2056 (86.13%) | 5437 (86.34%) |  |
| NSTEMI | 183 (7.67%) | 603 (9.58%) |  |
| STEMI | 99 (4.15%) | 140 (2.22%) |  |
| NYHA Classification, n (%) | | | 0.344 |
| Class I | 180 (7.55%) | 516 (8.19%) |  |
| Class II | 1982 (83.10%) | 5227 (83.01%) |  |
| Class III | 200 (8.39%) | 513 (8.15%) |  |
| Class IV | 23 (0.96%) | 41 (0.65%) |  |
| LVEF, % | 61.33 ± 10.56 | 61.29 ± 10.83 | 0.881 |

Data are shown as mean ± SD, median (IQR), or numbers (percentages).
